# Variations on Fibrinogen-Erythrocyte Interactions during Cell Aging

**DOI:** 10.1371/journal.pone.0018167

**Published:** 2011-03-28

**Authors:** Filomena A. Carvalho, Sofia de Oliveira, Teresa Freitas, Sónia Gonçalves, Nuno C. Santos

**Affiliations:** Instituto de Medicina Molecular, Faculdade de Medicina da Universidade de Lisboa, Lisbon, Portugal; University of Bergen, Norway

## Abstract

Erythrocyte hyperaggregation, a cardiovascular risk factor, is considered to be caused by an increase in plasma adhesion proteins, particularly fibrinogen. We have recently reported a specific binding between fibrinogen and an erythrocyte integrin receptor with a β_3_ or β_3_-like subunit. In this study we evaluate the influence of erythrocyte aging on the fibrinogen binding. By atomic force microscopy-based force spectroscopy measurements we found that increasing erythrocyte age, there is a decrease of the binding to fibrinogen by decreasing the frequency of its occurrence but not its force. This observation is reinforced by zeta-potential and fluorescence spectroscopy measurements. We conclude that upon erythrocyte aging the number of fibrinogen molecules bound to each cell decreases significantly, due to the progressive impairment of the specific fibrinogen-erythrocyte receptor interaction. Knowing that younger erythrocytes bind more to fibrinogen, we could presume that this population is the main contributor to the cardiovascular diseases associated with increased fibrinogen content in blood, which could disturb the blood flow. Our data also show that the sialic acids exposed on the erythrocyte membrane contribute for the interaction with fibrinogen, possibly by facilitating its binding to the erythrocyte membrane receptor.

## Introduction

Human erythrocytes (red blood cells) have a *in vivo* life span of approximately 120 days and are selectively removed from circulation via phagocytosis [1]. During its life span, the erythrocyte undergoes progressive physical and chemical changes, such as the decrease on cell volume with cell aging. This is presumably due to the loss of potassium and to the loss of membrane patches by microvesiculation, resulting in an increase on cell density [2]. Aged cells exhibit decreased deformability, electric mobility and lower surface negative charge [3], [4]. The membrane zeta-potential (which assesses the cell surface charge), together with the morphological and mechanical properties, are important structural and functional parameters of erythrocytes. They affect the deformability, and the circulation of erythrocytes in a blood vessel. Erythrocyte aggregation is also one of the most important factors affecting the blood flow. Increased erythrocyte aggregation is a cardiovascular risk factor, associated with hypertension, hypercholesterolemia and clinical conditions such as myocardial ischemia and thromboembolic states [5]. Hadengue *et al.* showed that in hypertension and hypercholesterolemia, the increase in erythrocyte aggregation could be attributed to an increase in the concentration of plasma fibrinogen. The prevailing hypothesis for the mechanism of fibrinogen-induced erythrocyte hyperaggregation was that it is caused by a nonspecific binding mechanism [6]. However, the published data on the changes in erythrocyte aggregation during hypertension pointed to the possible existence of other mechanism(s) [7].

The use of nanotechnologies for medical applications raises high expectations regarding diagnosis, drug delivery, gene therapy and tissue engineering. There is an increasing number of reports using AFM as a nanodiagnostic tool for patient cells. Beside its direct relevance on the identification of the fibrinogen receptor on erythrocytes and of a pharmacological strategy to inhibit it, our recent work was also a demonstration of the applicability and validation of the AFM-based force spectroscopy technique as a highly sensitive, rapid and low operation cost nanotool for the diagnostic and unbiased functional evaluation of the severity of hematological diseases arising from genetic mutations [8]. In this previous work, based on force spectroscopy measurements using an atomic force microscope (AFM), we reported the existence of a single-molecule interaction between fibrinogen and an unknown receptor on the erythrocyte membrane, with a lower but comparable affinity relative to platelet binding (average fibrinogen-erythrocyte and -platelet average (un)binding forces were 79 and 97 pN, respectively). The fibrinogen-platelet binding, essential for coagulation, depends on the platelet membrane receptor α_IIb_β_3_, an integrin. The receptor identified by us in erythrocytes is not as strongly influenced by calcium and eptifibatide (an α_IIb_β_3_ specific inhibitor) as the platelet receptor. However, its inhibition by eptifibatide indicates that it is an α_IIb_β_3_-related integrin. The results obtained for a Glanzmann thrombastenia (a rare hereditary bleeding disease caused by α_IIb_β_3_ deficiency) patient showed (for the first time) an impaired fibrinogen-erythrocyte binding. Correlation with genetic sequencing data demonstrated that one of the units of the fibrinogen receptor on erythrocytes is a product of the expression of the β_3_ gene, found to be mutated in this patient [8].

Knowing this, the purpose of the present study was to evaluate if fibrinogen-erythrocyte binding is dependent on *in vivo* cell aging, to assess the life span of the specific receptor and to get a further insight on its nature. Our data indicate that increasing erythrocytes aging, there is a significant decrease on the fibrinogen binding, by decreasing the frequency of its occurrence but not its binding strength. For the binding between fibrinogen and erythrocytes to occur, a lower fibrinogen concentration is needed on young erythrocytes than for the older ones.

## Methods

### Ethics Statement

Blood from healthy blood donors was obtained with their previous written informed consent, following a protocol with the Portuguese Blood Institute (Lisbon), approved by the Ethics Committee of the Faculty of Medicine of the University of Lisbon.

### Erythrocytes isolation

Blood was collected from adult donors into K_3_EDTA anticoagulant tubes. Total erythrocyte population was separated from the other blood components by centrifugation at 200 *g* for 15 min, at 20°C. After washing erythrocytes three times with NaCl 0.9% and centrifugation at 2000 *g* for 5 min, packed erythrocytes were resuspended in HEPES-buffered isotonic saline (NaCl 133 mM, KCl 4.5 mM and HEPES 10 mM, pH 7.4) to achieve a final concentration of 5×10^9^ cells/mL.

### Preparation of discontinuous Percoll gradients

Different erythrocytes subpopulations were isolated using a *Percoll* discontinuous gradient [9], [10], [11]. The gradient was built up in five layers containing 80% (0.360 mL), 74% (2.9 mL), 70% (2.9 mL), 66*%* (2.9 mL) and 60% (1.46 mL) *Percoll*, respectively, in HEPES buffer containing bovine serum albumin 5.25% (w/v). The density of the *Percoll* solutions varied between 1.087 and 1.098 g/mL [9]. An initial centrifugation of the gradient tube was done at 2700 *g*, for 30 min, at 20°C. 1.46 mL of the isolated erythrocyte suspension were layered on the top of the gradient tube and a new centrifugation was done at 2700 *g*, for 40 min, at 20°C, with a deceleration of 3 min. Erythrocyte fractions were collected by slowly pipetting over the liquid interface. The fraction containing the new erythrocytes population was concentrated in the upper layer (over *Percoll* 60% solution). Intermediate-aged erythrocytes population was collected over *Percoll* 66% and 70%, and the fraction containing the older senescent erythrocytes layered over the *Percoll* 74% solution. All the separated fractions were washed 3 times with HEPES-buffered isotonic saline at 2700 *g*, for 5 min, at 20°C.

The success of the age-dependent separation of erythrocytes into different fractions was confirmed by the determination of the sialic acid content on erythrocyte membranes, as previously reported [5], [9], [11], [12], [13].

### Preparation of erythrocyte integral membranes

Isolated erythrocyte integral membranes, commonly known as erythrocyte ghosts, were prepared from the isolated erythrocytes subpopulations. Packed erythrocytes were hemolyzed with 10 volumes of hypotonic 5P8 buffer (5 mM Na_2_HPO_4_, pH 8.0), followed by centrifugation at 12,000 *g* for 20 min to collect the membrane fraction [5]. The membrane pellets were washed with 5P8 buffer and centrifuged to remove hemoglobin from the membranes. Membrane protein concentration was determined on each sample with the Coomassie (Bradford) protein assay kit (Pierce Biotechnology, Inc., Rockford, IL) using bovine serum albumin as a standard.

### Determination of sialic acid content on erythrocytes subpopulations

Sialic acid content of isolated erythrocyte membranes was determined by the QuantiChrom™ Sialic Acid assay kit (BioAssay Systems, Hayward, CA). This assay is based on an improvement of the Warren method [14], in which sialic acid is oxidized to formylpyruvic acid, which reacts with thiobarbituric acid to form a pink colored product. It can be quantified at 549 nm and its absorbance is directly proportional to sialic acid concentration in the sample. This assay allows the determination of the free and total sialic acid concentrations on each sample, from which the concentration of membrane-bond sialic acid is calculated. We used a standard of N-acetylneuraminic acid (NANA) 10 mM to construct the calibration curve.

### Removal of sialic acid from erythrocytes membranes

After the separation of the total erythrocyte population from the other blood components, washed cells were resuspended at a concentration of 45% (v/v) in buffered saline glucose citrate (BSGC; 1.6 mM KH_2_PO_4_, 8.6 mM Na_2_HPO_4_, 120 mM NaCl, 13.6 mM sodium citrate, 11.1 mM glucose, pH 7.3), in the presence of 135 mUI/mL of neuraminidase (from *Clostridium perfringens*, type V, Sigma, St. Louis, MO), in order to promote the erythrocytes membrane sialic acids depletion. This suspension was incubated at 37°C for 1 h with gentle shaking and then washed three times with 10 volumes of BSGC buffer and centrifuged at 1500 *g* for 5 min. This sequence of washes of the cells is necessary to assure that neuraminidase is excluded from the cell buffer suspensions and subsequent experiments [5], [12].

### Atomic force microscopy samples preparation

For the atomic force microscopy studies, the three isolated erythrocytes subpopulations or the neuraminidase-treated erythrocytes were diluted (1/1000) with BSGC supplemented with calcium chloride 1 mM. Erythrocytes suspensions were placed on clean poly-L-lysine-coated glass slides and allowed to deposit, as previously described [8].

### AFM imaging

A NanoWizard II atomic force microscope (JPK Instruments, Berlin, Germany) mounted on the top of an Axiovert 200 inverted optical microscope (Carl Zeiss, Jena, Germany) was used for imaging and force spectroscopy experiments. The AFM head is equipped with a 15-µm z-range linearized piezoelectric scanner and an infrared laser. Imaging of erythrocytes was performed in air and in buffer, in tapping mode. Oxidized sharpened silicon tips with a tip radius of 6 nm, resonant frequency of about 300 kHz and spring constant of 40 N/m (ACT, Applied Nanostructures, CA) were used for the imaging. Imaging parameters were adjusted to minimize the force applied on the scanning of the topography of the cells. Scanning speed was optimized to 0.3 Hz and acquisition points were 512×512. Imaging data were analyzed with the JPK image processing v.3 (JPK Instruments).

### Fibrinogen-AFM tips functionalization

The protein-AFM tips functionalization process has been described elsewhere [8], [15], [16]. Briefly, cleaned AFM silicon nitride tips were silanized with 3-aminopropyl-triethoxysilane (APTES) for 1 h in argon atmosphere. Then the probes were treated with a glutaraldehyde solution 2.5% (v/v) for 20 min. Finally, the tips were placed in a 1 mg/mL human fibrinogen solution (Sigma) for 30 min at room temperature. The functionalized fibrinogen tips were immediately mounted on the AFM and used for the force spectroscopy experiments.

### Force spectroscopy on an atomic force microscope

Force spectroscopy measurements were performed using fibrinogen functionalized OMCL TR-400-type silicon nitride tips (Olympus, Tokyo, Japan), as previously described [8]. The spring constants of the tips were calibrated, resulting in values of 0.021±0.005 N/m. The applied force was adjusted to 1 nN before tip retraction. Data collection for each force-distance cycle was performed at 2 µm/s, leading to a loading rate of 4 nN/s. For any given experiment, approximately 15,000 force-distance curves were collected, analyzed and fitted to the worm-like-chain model (WLC) [17]. Each experiment was performed at least five times, each time on different blood samples and with different functionalized tips. The binning sizes chosen to build the force histograms consistently ranged between 4 and 6 pN.

### Labeling of human erythrocytes with di-8-ANEPPS

Once isolated by *Percoll* discontinuous gradient, each human erythrocytes subpopulation was labeled with the membrane dipole potential fluorescent probe di-8-ANEPPS (4-[2-[6-(dioctylamino)-2-naphthalenyl]ethenyl]-1-(3-sulfopropyl)-pyridinium) [18]. Briefly, a suspension at 1% hematocrit in HBSS (Hank's Balance Salt Solution, Invitrogen, Carlsbad, CA) buffer pH 7.4, supplemented with 0.05% (m/V) Pluronic F-127 (Sigma) and di-8-ANEPPS 10 mM (Molecular Probes, Invitrogen) was prepared with each erythrocyte subpopulation. The suspensions of erythrocytes with probe were incubated in the dark, at room temperature, with gentle agitation, for 1 h. Unbound di-8-ANEPPS was removed by two wash cycles, with centrifugations at 1500 *g*, for 5 min.

### Fluorescence spectroscopy measurements

The membrane probe di-8-ANEPPS assesses dipole potential by shifting its excitation spectrum upon a perturbation on this parameter (caused, in the present study, by the interaction of fibrinogen with the erythrocyte membrane). The experiments were conducted through the addition of different amounts of soluble human fibrinogen (0–3.4 mg/mL) to the di-8-ANEPPS labeled erythrocytes suspension (0.01% hematocrit). Differential spectra for detecting these shifts are obtained by subtracting the excitation spectrum of labeled cells in the presence of each of the different fibrinogen concentrations from the spectrum in its absence. Before subtraction the spectra were normalized to the integrated areas to reflect only spectral shifts. The differential spectra are waveform shaped, which amplitude directly correlates with the peak shifting magnitude, and hence, with the dipole potential variation.

To define the dipole potential changes due the spectral shift, a ratio was established from the fluorescence intensities at two wavelengths on the sides of excitation spectrum peak [19]. We chose them by taking the corresponding wavelength values for the minimum and the maximum of the differential spectra, defining the ratio *R* for this case as the ratio between the fluorescence intensities obtained with the excitation wavelength at 455 nm and 525 nm, keeping the emission wavelength constant at 670 nm. A decrease in the membrane dipole potential leads to a red-shift in the membrane incorporated di-8-ANEPPS and, consequently, to a decrease on this ratio [19]. The variation of *R* with the fibrinogen concentration was calculated, plotted and analyzed by a single binding site model [19], following the equation:
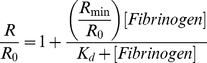
(1)
*R* values are normalized dividing by *R*_0_, corresponding to zero fibrinogen concentration. *R*_min_ defines the asymptotic minimum value of *R* and *K_d_* is the equilibrium dissociation constant.

All the fluorescence spectroscopy measurements were made in a Varian Cary Eclipse (Mulgrave, Australia) fluorescence spectrophotometer. Excitation and emission bandwidths were 5 nm and 10 nm, respectively, on all measurements.

### Zeta-potential samples preparation

The samples obtained for each erythrocytes subpopulation were set to 0.035% hematocrit (kept constant during the experiments) in HBSS buffer pH 7.4, in the absence and presence of different soluble human fibrinogen concentrations (0–4 mg/mL). The suspensions were filtered using a syringe filter with 0.45 µm pore size (Whatman, Florham Park, NJ) to remove any large scattering particle, which would bias the light scattering measurements.

### Zeta-potential measurements

Measurements were conducted on a dynamic light scattering and zeta-potential equipment Malvern Zetasizer Nano ZS (Malvern, UK), equipped with a He-Ne laser (λ = 632.8 nm). The zeta-potential (ζ) of the samples were determined, at 25°C, from the mean of 15 measurements, with 60 runs each, with an applied potential of 30 V, in the absence and presence of different soluble human fibrinogen concentrations, using disposable zeta cells with platinum gold-coated electrodes (Malvern). For a recent review on zeta-potential measurements, and their biochemical and biophysical applications, see reference [20]. The electrophoretic mobility obtained was used for the zeta-potential calculation through the Smoluchowski equation [21],
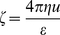
(2)where *u* represents the electrophoretic mobility, *η* the viscosity of the solvent and *ε* its dielectric constant. The variation of the zeta-potential (Δζ) for each sample was calculated by subtracting from the zeta-potential value of the sample the initial value corresponding to zero fibrinogen concentration. These differences can be plotted as a function of fibrinogen concentration, and the experimental data fitted using the equation [22], [23]:
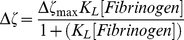
(3)where Δζ_max_ is the maximum amplitude of variation of the zeta-potential induced by the interaction with fibrinogen, and *K_L_* corresponds to the inverse of the value of fibrinogen concentration at ζ_max_/2.

### Statistical analysis

Unpaired Student's *t*-test was used for statistical analysis of intergroup comparison. Differences were considered statistically significant for p<0.05.

## Results

### AFM imaging and force spectroscopy of fibrinogen-erythrocyte subpopulations

**Figure 1:**
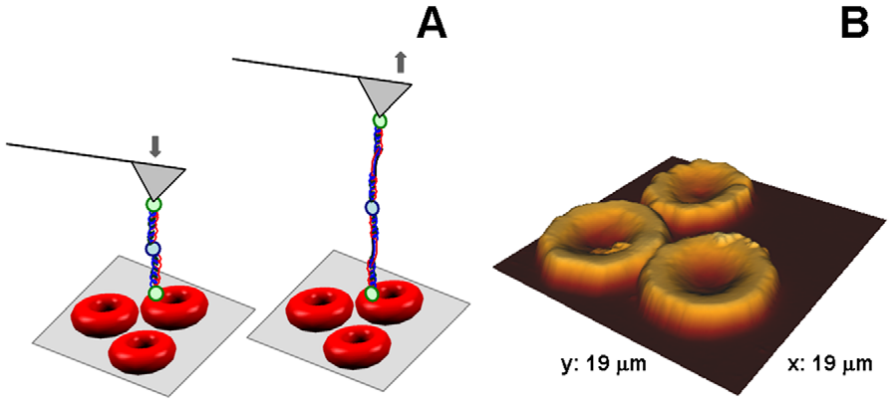
Force Spectroscopy technique at an Atomic Force Microscope. (A) Schematic representation of the erythrocytes deposited on a poly-L-lysine-coated glass slide and an AFM tip chemically functionalized with fibrinogen molecule(s). The arrows represent the approach and retract cycles during the force spectroscopy measurements. When approaching the tip to the sample, the fibrinogen molecule may contact with cell receptor(s) and the binding between them can occur. By retracting the tip away from the sample, the binding force necessary to break this bond, at the single-molecule level, can be measured. (B) Air tapping-mode AFM images of typical circular, biconcave human erythrocytes, from healthy donors, deposited on poly-L-lysine coated glass slides (height 3D image).

When performing force spectroscopy measurements (*vd*. Fig. 1*A*), the force *vs*. distance curves acquired after the fibrinogen-functionalized AFM tips contact with the erythrocytes surface (imaged in Fig. 1*B*) show well-defined and measurable adhesion forces. AFM imaging revealed the common disk shape of human erythrocytes, with 8–10 µm of diameter and a height of approximately 900 nm. The cells showed good adherence to poly-L-lysine (a positively charged polymer) coated glass slides with the experimental conditions described.

**Figure 2:**
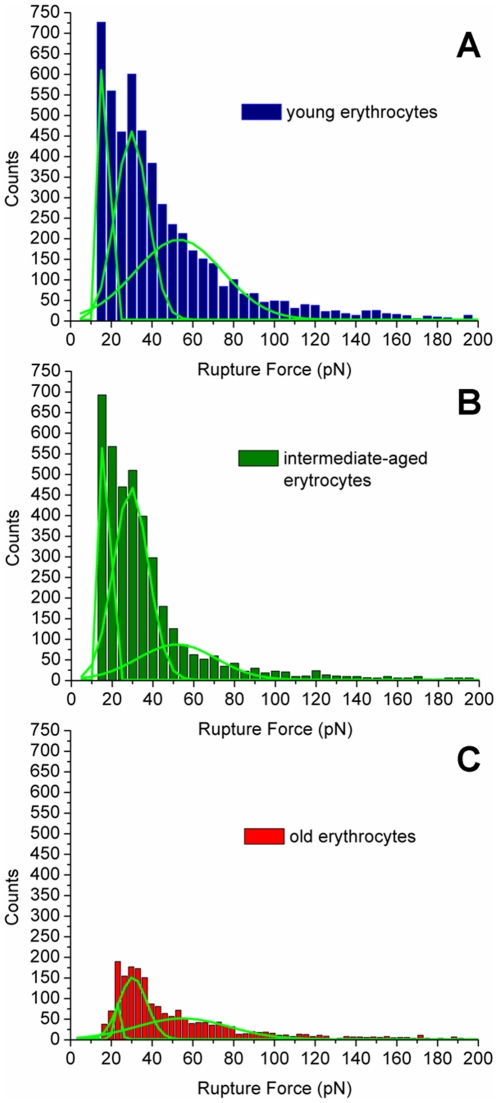
Rupture-force histograms of fibrinogen-erythrocytes interactions. Rupture-force histograms from about 15,000 force measurements for each of the interactions between fibrinogen and young erythrocytes (blue), intermediate-aged erythrocytes (green) and old erythrocytes (red) subpopulations. All measurements were done with an applied force of 1 nN, pulling speed of 2 µm/s and loading rate of 4 nN/s. Gaussian peaks adjustment curves are shown in light green.

The repeated measurement of the adhesion events allows us to create rupture-force histograms for each erythrocyte population studied (*vd*. Fig. 2). Rupture force values are defined as the force necessary to break the bond between a single fibrinogen molecule and an erythrocyte receptor, which is characterized by the instantaneous jumps in force observed on force-distance curves (data not shown). Experimental data were fitted with Gaussian curves to obtain the average rupture force for a single fibrinogen-cell receptor binding, yielding values of 17±6 pN for young erythrocytes, 17±4 pN for intermediate-aged erythrocytes and 22±0.3 pN for old erythrocytes. The differences between these values are not statistically significant. The two peaks observed on each histogram with forces above 30 pN are probably from multiple binding events between different fibrinogen molecules and its cell receptor.

Each rupture-force histogram shown on Fig. 2 was adjusted to a maximum of 750 counts on the yy-axis. We can observe from Fig. 2 that old erythrocytes have a significant decrease on the frequency of the binding to fibrinogen molecules, despite the fact that the few observed adhesions have identical rupture forces to those achieved with young or intermediate-aged erythrocytes populations. The frequency of adhesion-rupture events achieved for the young erythrocytes-fibrinogen system was 18.6%. For the intermediate-aged erythrocytes-fibrinogen system this percentage was slightly lower (13.2%) and for old erythrocytes population the value significantly decrease (4.6%). From these adhesion events, approximately 75% were of single rupture events and the remaining 25% were from double or multiple steps binding events, for the three studied erythrocytes populations.

### Variation on the erythrocyte membranes sialic acid content upon aging

As the best control of the age-dependent separation of erythrocytes into different fractions, we determined the concentration of sialic acid on each cell subpopulation. The achieved values are of the same order as previously reported by others [5], [12]. We obtained a decrease of the sialic acid concentration on erythrocytes membranes of 22.5% from young to old erythrocytes. This result is in agreement with the ones obtained by other authors, who reported a percentage of removal of sialic acids from young to old erythrocytes of 21.2% [11] and of 15% [13]. This loss of membrane sialic acid was also previously documented by several other authors [3], [5], [12].

### Neuraminidase effect on erythrocytes–fibrinogen binding

Force spectroscopy studies were also performed with neuraminidase-treated erythrocytes to evaluate the effect of the presence of sialic acids on erythrocyte membrane on the binding to fibrinogen molecules. The achieved force rupture histogram shows an average fibrinogen-erythrocyte binding force of 62 pN, which is slightly lower than the obtained with the untreated erythrocytes (79 pN for total erythrocytes population). Remarkably, the percentage of (un)binding events decreased significantly, from 17% to 1.9%. These events can be termed (un)binding events because, during the force spectroscopy approach/retraction cycles, each time that there is an adhesion/binding event between a fibrinogen molecule and a erythrocyte receptor, its binding force is in fact measured by the changes on the AFM tip deflection at the retraction curve when bond break occurs.

### Fibrinogen-erythrocyte binding evaluation by fluorescence spectroscopy measurements

**Figure 3:**
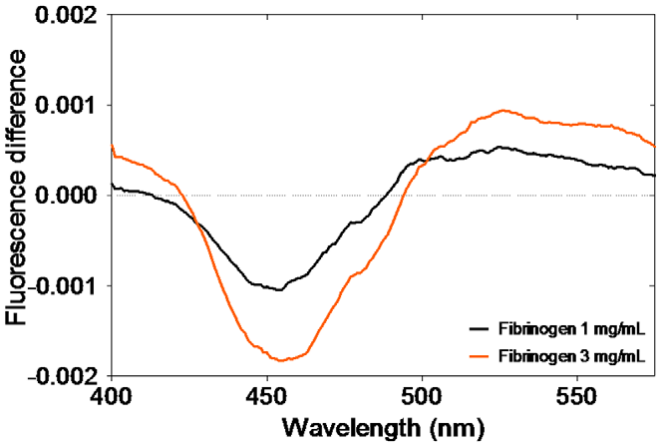
Differential spectra of di-8-ANEPPS-labeled erythrocytes. Differential spectra of di-8-ANEPPS-labeled total erythrocytes in the presence of 1 mg/mL (black) and 3 mg/mL (orange) of fibrinogen (after subtraction of the spectrum obtained in the absence of fibrinogen). The shape of the spectra shows a spectral shift to the red, indicative of a decrease in membrane dipole potential. The corresponding wavelength values for minimum and the maximum of the differential spectra defined the ratio *R* I_455nm_/I_525nm_ was used for further experiments.

**Figure 4:**
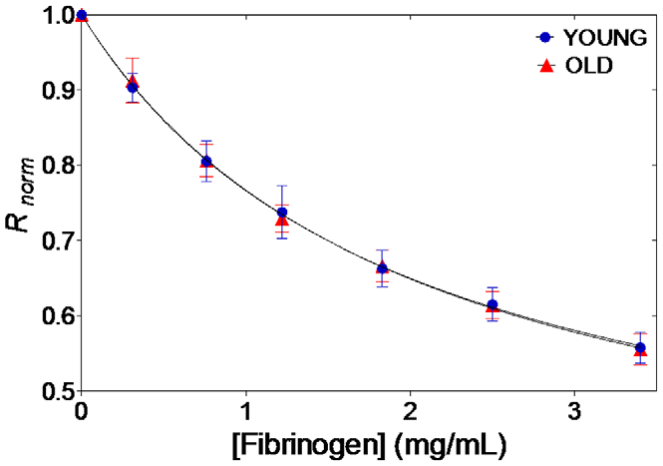
Fibrinogen affinity to different erythrocytes subpopulations. The dependence of the ratios obtained for young and old erythrocytes for different fibrinogen concentrations were analyzed by a single binding site model (solid line) in order to quantify the dissociation constants. Plotted values correspond to ratio values normalized by the value corresponding to the absence of fibrinogen.

The interaction of fibrinogen with the different erythrocytes subpopulations was assessed by fluorescence spectroscopy, using the dipole potential membrane probe di-8-ANEPPS [18], [19], [24]. This probe indirectly allows the quantitative assessment of the binding of fibrinogen to erythrocytes, based on the changes on the cell membrane potential arising from the fibrinogen binding. The differential fluorescence excitation spectra obtained for two fibrinogen concentrations, using labeled total erythrocytes, are shown in Fig. 3. The minimum on the fluorescence difference curve obtained at low wavelengths indicates a decrease in the dipole potential of the membrane upon fibrinogen binding. A detailed study of the interaction was obtained by plotting the normalized parameter *R* as a function of fibrinogen concentration (Fig. 4). As fibrinogen concentration increases, the ratio *R*_norm_ diminishes in a fibrinogen concentration-dependent manner. The fitting of the data to a single binding site model yields *K_d_* values of (58±2)×10^−7^ M and (60±3)×10^−7^ M for young and old erythrocytes, respectively. This variation is not statistically significant.

### Fibrinogen-erythrocyte binding evaluation by zeta-potential measurements

**Figure 5:**
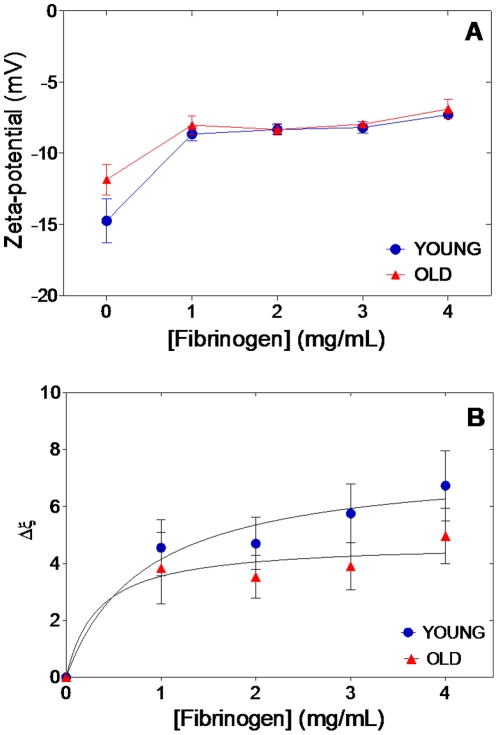
Zeta-potential of erythrocytes populations. (A) Zeta-potential values of isolated young and old erythrocytes in the presence of different fibrinogen concentrations. (B) Variation of zeta-potential for the same erythrocyte subpopulations. Δζ_max young_ = (7.6±1.8) mV; Δζ_max old_ = (4.7±1.1) mV. Statistically significant differences (p<0.05) were found between the two erythrocytes subpopulations.

The zeta-potential values obtained for young and old erythrocytes in the absence of fibrinogen clearly show the difference between both subpopulations (*vd*. Fig. 5*A*). In the absence of fibrinogen, young erythrocytes have significantly lower (p<0.05) zeta-potential values (−14.8 mV) when compared to the old erythrocytes subpopulation (−11.3 mV). In the presence of fibrinogen, there is a substantial increase of the zeta-potential for both subpopulations, converging to a similar threshold (*vd*. Fig. 5*A*). The electrophoretic mobility and zeta-potential values can be related with the affinity of the interaction. To measure the extension of the fibrinogen binding to the two erythrocyte subpopulations, the Δζ values were calculated and fitted to Eq. 3 (Fig. 5*B*). The extension of the fibrinogen-young erythrocytes cells interaction obtained, Δζ_max_, is (7.6±1.8) mV, significantly higher than the value of (4.7±1.1) mV, obtained for old erythrocytes.

## Discussion

In this study, we evaluated if the binding between fibrinogen and erythrocytes is dependent on the *in vivo* process of cell aging. Using force spectroscopy, at an atomic force microscope, zeta-potential and fluorescence spectroscopy measurements, we could conclude that with the increase of the age of erythrocytes, a significant decrease of the binding to fibrinogen occurs.

We recently reported the existence of a specific binding between fibrinogen and an erythrocyte receptor with a (un)binding force lower but comparable to the determined for the fibrinogen-platelet binding [8]. We also showed that this interaction is mediated by an erythrocyte integrin α_IIb_β_3_-like receptor, in which one of its units is expressed by the same gene as platelet's β_3_ unit. Now, also by force spectroscopy technique, and after the isolation of erythrocyte subpopulations with different ages, the (un)binding force values obtained were similar for the three subpopulations tested. It should be mentioned that the values obtained were lower than those achieved with the total erythrocyte population (79 pN [8]). We hypothesize that this discrepancy could be explained by the presence of bovine serum albumin on the *Percoll* gradient cells isolation isotonic buffer. Bovine serum albumin is known to have capacity of binding to lipids so it could bind to the erythrocyte phospholipid membrane, coating part of the cell surface and make the fibrinogen receptors on erythrocyte membrane less accessible for the binding to fibrinogen [25], [26]. Therefore, force results obtained after Percoll isolation should only be correctly compared quantitatively with those obtained in the same medium.

Force spectroscopy reveals to be a good biophysical method to determine the interaction forces between fibrinogen and human blood cells. At variance with other methodologies, the process of cell isolation is not an issue with this technique because the measurements are conducted at the single-cell level. After isolating the erythrocytes, the fibrinogen interactions are measured on the top of a single erythrocyte at a time, while it can be optically imaged in real-time, assuring that it is not another type of blood cell. The possibility of the AFM-based force spectroscopy measurements with erythrocytes being an artifact due to platelet membrane fragments bound onto erythrocytes surface can be ruled out based on the following evidences: *i*) considering the binding/unbinding frequencies obtained on this study and on our previous results [8], for this hypothesis to be possible it would be necessary that at least 80% of the membranes of all erythrocytes would be covered by “plastered” fragments; *ii*) it would also be necessary that the platelet fragments-erythrocytes binding would be considerably stronger than the fibrinogen-receptor interaction, in order for the fragments not to be pulled-off from the erythrocytes membrane upon AFM tip retraction; and, *iii*) our previous kinetics, thermodynamics, calcium-dependence and inhibition by eptifibatide results [8] show that the fibrinogen-receptor in erythrocytes is different from the receptor in platelets.

On the AFM imaging of the different erythrocytes populations, we did not find considerable morphological changes, which obviously do not exclude possible changes on other cell properties. As assessed by force spectroscopy, there were also no significant changes on the rupture force value of the three studied erythrocytes populations. However, we obtained a dramatic decrease on the percentage of binding events with erythrocyte aging. There are several reports studying the altered properties of erythrocytes during the senescence process [2], [3], [27]. During erythrocytes aging, there is an increase on the cell density, decreases on deformability and electric mobility, and a lower negative membrane charge [2]. Activities of some cytoplasmatic enzymes also decline during erythrocyte aging. An alteration in the lipid composition (phospholipids, unesterefied cholesterol and glycolipids) and loss of asymmetry between the inner and outer leaflets of the lipid bilayer is associated with erythrocytes senescence. The interaction between phosphatidylserine and the cytoskeleton component spectrin triggers the accumulation of large amounts of phosphatidylserine and phosphatidylethanolamine on the outer leaflet erythrocyte membrane [3]. Other changes in membrane components and organization are also observed on aged erythrocytes, including changes on carbohydrates (exposure of β-galactosyl residues that are recognized by lectin-like receptors), proteins (e.g., modification of band 3 protein, a major transmembrane glycoprotein in erythrocyte, either by proteolytic cleavage or aggregation) and decrease on the activities of several enzymes (including enzymes of the glycolytic and pentose shunt pathways, involved in cell thiol metabolism and protection against oxidative damage, enzymes involved in the catabolism of nucleotides, and others) [2], [3].

In spite of the success on the assessment of the fibrinogen-erythrocyte interaction through the changes in the dipole potential of the labeled membranes, we did not find a statistically significant difference between the fibrinogen dissociation constants obtained for young and old erythrocytes. However, the values obtained indicate a higher binding of fibrinogen to younger erythrocytes, pointing to the same conclusion as those obtained by force spectroscopy. Several studies have been developed to clarify the behavior of fibrinogen in the presence of ligands of physiological importance [28], [29]. A previous work indicated that β-estradiol binds to human fibrinogen with a *K_d_* = 1.55×10^−7^ M [28]. By comparing the dissociation constant obtained in our experiments with those obtained in previous studies, we can say that it corresponds to a high affinity binding site [28], [29].

Isolated fractions of old erythrocytes move more slower in the electric field than young erythrocytes of the same blood sample [30], [31]. In order to analyze the variation of the electrostatic forces for erythrocytes subpopulations in the absence and presence of fibrinogen, the interaction was monitored by zeta-potential measurements. In agreement with the results obtained AFM-based force spectroscopy measurements (and with the fluorescence data, if they would be statistically-significant), two different behaviors were registered by zeta-potential measurements for young and old erythrocytes, clearly showing the difference between both subpopulations (*vd*. Fig. 5*A*). Furthermore, our data show that the zeta-potential, or the related electrophoretic mobility, can be used to determine the extension of the interaction between two particles, by calculating the value of Δζ_max_. The obtained results clearly reflect the behavior of both cell types in the presence of different fibrinogen concentrations. The extension of fibrinogen-young erythrocytes cells interaction obtained is estimated by Δζ_max_ = (7.6±1.8) mV, a value significantly higher than the obtained for old erythrocytes, Δζ_max_ = (4.7±1.1) mV.

In the absence of fibrinogen, young erythrocytes have significantly lower values of zeta-potential (−14.8 mV) when compare to the old erythrocytes subpopulation (−11.3 mV), in agreement with the reported by other authors [27]. The 24% decreased on the surface charge from young to old cells may be explained by the fact that sialic acid, the principal source of membrane negative charge on erythrocytes, is lost during cell aging (*vd*. results of sialic acid concentrations on different aging erythrocyte subpopulations), reducing the total surface negative charge [27], [32]. By increasing fibrinogen concentration to 4 mg/mL, the zeta-potential threshold attained is almost identical for both sample types, showing that the charge in the erythrocyte surface is partially neutralized by the presence of fibrinogen. These results clearly reinforce the hypothesis that the electrostatics forces contribute for the first step of fibrinogen-erythrocyte interaction [29], facilitating the binding to the fibrinogen membrane receptor. However, this convergence for the same charge value, away from complete neutralization, despite the difference in the initial membrane charge, suggests that the key parameter for the interaction is not the global membrane charge, but the presence of a specific charged membrane component, that would be progressively lost upon cell aging. This observation prompted us to evaluate if the membrane sialic acids would be this charged component, by testing the effect of the neuraminidase-promoted sialic acids depletion. The force spectroscopy data obtained after the treatment with neuraminidase indicate that sialic acids can be this specific charged membrane component, being progressively lost during erythrocyte aging.

The observed decrease of membrane sialic acids content may be associated with an alteration of glycophorin A, a major sialoglycoprotein of human erythrocyte membrane, which may contribute to erythrocytes transient aggregation in circulation [5], [33]. Removal of sialic acid residues of the saccharide chains of glycophorin A, during erythrocyte aging, results in the exposure of a new antigen, recognized by immunoglobins G, leading to a normal process of elimination of aged cells by macrophages [5]. As we found that removal of sialic acids decreases the number of fibrinogen-erythrocyte receptor binding events, without significantly affecting the (un)binding forces of each interaction at the single-molecule level, we can presume that the existence of erythrocyte membrane-bounded sialic acids facilitate the binding of the cell to fibrinogen molecules, by increasing the probability of the contact between fibrinogen and its erythrocyte membrane receptor.

Using a fluorescence spectroscopy methodology, we did not find a significant difference between the fibrinogen specific affinities to the different erythrocytes population. On the other hand, significant changes in rupture force and in the fibrinogen-dependent zeta-potential variation were found during erythrocyte aging. As we have pointed out before [8], some methods are able to show that the fibrinogen-erythrocyte interaction exists, but fail on further characterizing it. This is probably results from the transient character of the fibrinogen-erythrocyte interaction, which can be properly studied mainly by force spectroscopy and also by zeta-potential measurements, but limits the details that can be assessed by fluorescent membrane dipole potential probes. This transient interaction cannot lead to clot formation, but is able to increase erythrocyte aggregation, increasing cardiovascular risk. We have previously shown that, in some situations, the lifetime of the fibrinogen-erythrocyte bond is only 0.04% of the lifetime of the fibrinogen-platelet binding [8], pointing out why only some methodologies can properly follow subtle variations on the binding of fibrinogen to erythrocytes.

The present study indicates that the fibrinogen-erythrocyte receptor binding can be lost, masked or progressively turned to non-functional with the *in vivo* erythrocyte senescence process. Additionally, our data points out to the role of the sialic acid residues, present in several erythrocyte membrane components, as promoters of the interaction of fibrinogen with its receptor. Therefore, the decrease on the fibrinogen-erythrocyte interaction during cell aging can be largely associated to the progressive depletion of sialic acids on older erythrocytes. Knowing that younger erythrocytes bind more to fibrinogen, we could presume that this population is the main responsible for some cardiovascular diseases associated with an increase on the fibrinogen content in blood, which could disturb its normal flow.
